# Reduced processing of afforded actions while observing mental content as ongoing mental phenomena

**DOI:** 10.1038/s41598-024-60934-6

**Published:** 2024-05-02

**Authors:** Sucharit Katyal, Oussama Abdoun, Hugues Mounier, Antoine Lutz

**Affiliations:** 1 EDUWELL Team, Lyon Neuroscience Research Centre, INSERM U1028, CNRS UMR5292, Lyon 1 University, Lyon, France; 2 Department of Psychology, University of Copenhagen, Copenhagen, Denmark; 3 L2S - Laboratoire des signaux et systemes, Université Paris-Saclay, CentraleSupélec, CNRS, Gif Sur Yvette, France

**Keywords:** Cognitive neuroscience, Human behaviour

## Abstract

While consciousness is typically considered equivalent to mental contents, certain meditation practices—including open monitoring (OM)—are said to enable a unique conscious state where meditators can experience mental content from a de-reified perspective as “ongoing phenomena.” Phenomenologically, such a state is considered as reduction of intentionality, the mental act upon mental content. We hypothesised that this de-reified state would be characterised by reduced mental actional processing of affording objects. We recruited two groups of participants, meditators with long-term experience in cultivating a de-reified state, and demographically-matched novice meditators. Participants performed a task with images in two configurations—where objects did (high-affordance) and did not imply actions (low-affordance)—following both a baseline and OM-induced de-reified state, along with EEG recordings. While long-term meditators exhibited preferential processing of high-affordance images compared to low-affordance images during baseline, such an effect was abolished during the OM state, as hypothesised. For novices, however, the high-affordance configuration was preferred over the low-affordance one both during baseline and OM. Perceptual durations of objects across conditions positively correlated with the degree of µ-rhythm desynchronization, indicating that neural processing of affordance impacted perceptual awareness. Our results indicate that OM styles of meditation may help in mentally decoupling otherwise automatic cognitive processing of mental actions by affording objects.

## Introduction

Long-term mental training through meditation has been proposed to promote a distinct phenomenological way of consciously experiencing the world, where a meditator can cultivate a meta-perspective on mental contents, as ongoing phenomena constituted within consciousness. This has been labelled decentring^1^, cognitive defusion^2^, mindful attention^3^, dereification^4^, or opacification^5^, and phenomenological^6^ or experiential^7^ reduction. For example, dereification—i.e., “*… the degree to which thoughts, feelings, and perceptions are phenomenally interpreted as mental processes rather than as accurate depictions of reality*” – is viewed as a key process associated with mindfulness meditation^4^. Dereification is particularly targeted in a style of mindfulness meditation called open monitoring (OM)^8^, as well as a more advanced form of OM, called open presence^9^. It involves downregulating automatic cognitive and emotional interpretations of sensory and perceptual stimuli. Previous studies have shown that de-reified states can involve downregulation of affective processes like food cravings accompanying appetitive food images^3,10,11^. Currently, little is known about the effect of dereification on purely perceptual processes that accompany sensory such as affordance of objects.

In his ecological approach to perception, Gibson coined the idea of ‘affordances’^12^, or what an environment ‘offers’ an organism with regards to how they respond to it. Cooper elaborates on affordance as follows^13^: “*An affordance is an action that is suggested or somehow implied to an agent capable of performing that action by an object or situation in the agent’s immediate environment. Thus,… a cup with a handle in reach might be said to afford grasping by the handle with a certain type of grip*.” From a cognitive standpoint, an action afforded by an object in the environment would lead to automatic planning processes about how the individual could act on it^14^. Indeed, research has shown that individuals perceiving objects that afford actions exhibit supplementary brain and behavioural signatures of action processing, and that such processing can occur largely automatically (involuntarily) and outside of the conscious awareness (for review, see^15^). Moreover, automatic action processing of object affordances occurs not only for real objects but also images of such objects^16,17^. In other words, when seeing images of objects, individuals tend to naturally “reify” such objects (i.e., perceive images of objects as if they were real). A parallel body of research however has also shown that such automatic mental process of affordances can be mitigated by attention—for example, by diverting attention to non-action-related features or spatial locations^18–20^. The question we attempt to address in this study is whether automatic mental processing afforded by images of objects can moreover be mitigated by means of meditative dereification, a process/ability that would be more strongly developed in individuals with long-term meditation training compared to novice meditators.

**Figure 1:**
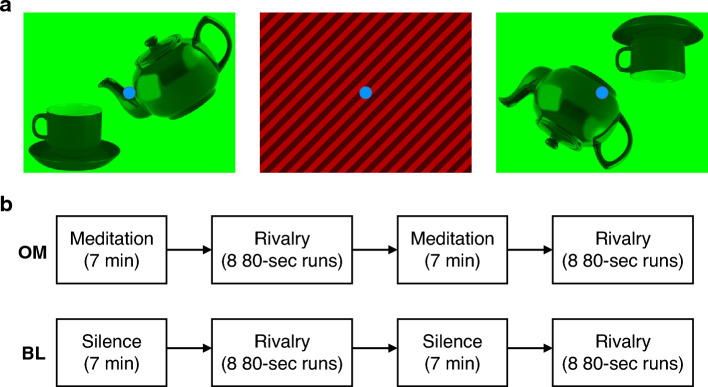
(**A**) One of the two pairs of stimuli used in our study. For a particular run, we presented the red grating image in one eye (middle) and a green image with objects in the other eye (colours counterbalanced across eyes over the runs). In one half of the runs, the object image was in a high-affordance configuration (left) where the two objects were oriented towards each other in a way that implied action. In the other half of the runs, the objects were in a low-affordance configuration (right) where the same objects were oriented in a way that did not imply action. Participants were instructed to respond if they saw the red image, the green image or a mixture of the images. (**B**) The study involved two blocks, baseline (BL) and open monitoring mindfulness (OM). The blocks were counterbalanced across participants within both groups. For the BL and OM blocks there were two sub-blocks of eight 80-s binocular rivalry runs preceded by a 7-min silence and meditation period respectively.

One way in which automatic mental processing of action-related stimuli is studied in the lab is by testing brain and behavioural responses to images of everyday tools and objects that afford actions. As a control condition, these images are compared to the same tools and objects in a configuration that do not afford an action. For example, an upright teapot tilted and oriented towards a cup would afford the action of the teacup being held as if pouring tea into the cup, whereas an inverted teapot facing away from an inverted teacup would not (Fig. 1)^16^. In these experiments, images that afford actions are found to undergo preferential processing compared to the ones where action is not afforded^15^. This preferential processing can be observed as reduced visual extinction in neuropsychological patients^16^, better accuracy^21^ and faster reaction times^17^ for images with higher affordance.

Another way preferential action-related vs. non-action-related processing has been studied is by measuring its effect on visual processing using interocular suppression paradigms like binocular rivalry^22,23^. Binocular rivalry is a perceptual phenomenon where individuals are shown two separate images to the two eyes, which results in their perception alternating between the two images every few seconds. Here, if all other visual features of images are controlled, the duration for which an image remains perceptually dominant on average can quantify the degree of preferential processing of that image compared to another image. For example, Pace and Saracini^23^ presented action-related images to one eye and a neutral image (chequered boxes) to the other eye of participants. Instead of manipulating action in the image itself, they instructed participants in one condition to mimic the action in the image with their hands, and in a second condition to not mimic the action. They found that the dominance durations of the action image compared to a neutral image was longer when the participants mimicked the action. Their results indicate that actions lead to preferential processing of action-related visual stimuli.

In addition to behaviour, action-related processing has also been observed in brain activity. A classical signature associated with physical actions is desynchronisation of the *µ* rhythm (8–12 Hz) over somatomotor cortices^24–26^. Much like its occipital counterpart, alpha (7–13 Hz), *µ* is a suppressive rhythm. It is strong in the absence of somatomotor activity and desynchronises when one engages in such activity. Interestingly, *µ* desynchronisation is observed not only during physical actions, but also during imagined^27^ and observed actions^28^. It has therefore been suggested as a brain correlate of action affordance or automatic motor planning^26,29^. Moreover, desynchronisation of *µ* rhythm is observed not only for stimuli that are perceived consciously but also ones suppressed from consciousness through binocular rivalry^30^, supporting the idea that affordance processing in the brain can even occur outside of conscious awareness.

Finally, meditation training is proposed to not only enable de-reification behaviourally^3,10^, but also experientially in a manner that allows a meditator to become metacognitively aware of such dereification^4,31^. In other words, not only would we expect a trained meditator to have the ability to reduce automatic action processing when viewing a sensory stimulus that implies an action, but also potentially possess introspective access into the degree of de-reification. It awaits to be determined whether the introspectively determined degree of de-reification matches with behavioural and neural signatures of automatic action processing.

In the present study, we test the idea that long-term meditators (LTM) can assume a dereified mental stance from which they can suspend or attenuate action-related processing when viewing images that afford actions, using behavioural, neural and introspective measures. In contrast, we expected that novice meditators (NVM) would not be able to enter such a stance (or do so to a lesser degree compared to LTM). To test this idea, we administered the binocular rivalry task to LTMs and NVMs where we manipulated implied action (affordance) in the visual stimuli while measuring participants’ EEG. We used a binocular rivalry task to quantify preferential affordance processing instead of a performance-based measure like accuracy or reaction time. This was because maintenance of a dereified mental stance is said to involve a type of “veridical” sensory perception that is devoid of expectations and outcomes, which would potentially be disrupted by having participants engage in a task where they would be trying to maximise their performance. Moreover, during a binocular rivalry task block can be administered to participants for a prolonged period where they report their perceptual alternations. This would offer fewer disruptions to participants’ meditative state compared to tasks that require repeated presentation of many shorter trials interspersed with inter-trial intervals.

During the binocular rivalry task, the participants were shown an image displaying objects in one eye and a diagonal grating in the other eye. The image with objects could be in one of two configurations counterbalanced across runs: a high-affordance configuration where two objects afforded an action, and a low-affordance condition where the same objects were inverted and thus did not afford action processing to the same extent as the high-affordance configuration (Fig. 1). The binocular rivalry task was administered to each participant in two blocks, one following a baseline (BL) period where participants kept their eyes passively closed and another following a period of open monitoring (OM) meditation. Participants were further instructed that following the meditation period they continue with the OM meditative “attitude” during the task. After each binocular rivalry run participants provided self-reports on how “subjectively real” they experienced the object images to be during that run.

As a behavioural manipulation of affordance, we expected that the ratio of the mean duration for which the participants reported seeing the high-affordance image to the neutral grating image (bHAR) would be significantly greater than the ratio of the mean duration for the low-affordance image to the grating image (bLAR) during baseline (bHAR > bLAR)^23^. We further hypothesised that, compared to baseline, during the meditative state, the difference between bHAR and bLAR would be significantly reduced in LTM but not in NVM. As a neural correlate of affordance manipulation, we compared *µ* rhythm on runs where participants were shown the high- vs. low-affordance image as the object image (we did not distinguish whether the object image was perceptually dominant, or not, because µ desynchronisation has been observed for both dominant and suppressed images during interocular rivalry^30^). Here, we expected that the power of *µ* rhythm on runs when participants were presented with the high-affordance image (mHA) would be lower than *µ* power when participants were shown the low-affordance image (mHA < mLA). And compared to baseline, during the meditative state, mLA—mHA would be reduced in the LTM but not in NVM. Finally, with respect to self-reports, we hypothesised that participants would report experiencing the high-affordance images (sHA) to be more subjectively real than the low-affordance (sLA) ones (sHA > sLA) and following meditation sHA—sLA would significantly reduce in LTM but not in NVM.

## Materials and methods

### Participants

Nineteen long-term meditation practitioners and twenty-one novice meditators participated in the study, as part of the Brain & Mindfulness project (for details of the overall protocol see^32^). All participants were naïve to the binocular rivalry paradigm. Novice participants did not have significant experience with meditation or other mind–body training techniques prior to recruitment. Instead, they received a 2-day meditation training in the lab. Long-term meditators on the other hand had expertise in the open monitoring or open presence (see below for differences) styles of meditation. While there are currently no standardised criteria for classifying meditation expertise, here we operationalized it by combining objective and intersubjective criteria. Firstly, practitioners had to have learnt and practised the same styles of meditations, as it is proposed in traditional 3-year meditation retreat in the Kagyu or Nyingma school of Tibetan Buddhism. They also had to have a minimum requirement of 10,000 h of formal practice as well as a regular daily practice Secondly, a research assistant (CB) highly familiar with these practices checked that the practitioners’ Buddhist communities perceived them as meditators sufficiently experienced in these practices. Recruited participants reported no use of psychoactive medication, history of neurological or psychiatric illness and had normal or corrected-to-normal visual acuity. Participants provided written informed consent prior to the study and were paid for participation. All procedures were performed according to the guidelines and regulations in ethics approval on human research obtained from CPP Sud-Est IV (2015-A01472-47).

### Stimulus and paradigm

Experimental stimuli were generated using the Psychophysics toolbox (PTB–3)^33^ in MATLAB (The MathWorks Inc., MA), and were presented on a 24″ monitor (Acer KG241PBMIDPX) placed 52 cm from the participants’ eyes. A custom-built mirror stereoscope was used to present dichoptic stimuli. Visual stimuli were presented within a rectangular aperture of 5.75° × 5.50° of visual angle.

Before beginning the experiment, each participant was trained to report binocular rivalry alternations over four 80-s training and practice runs where participants adjusted the stereoscope and learnt to report binocular rivalry alternations with a keypad.

The binocular rivalry task was designed with three main factors of interest, *group* (long-term meditators (LTM), novices (NVM)), *affordance* (high, low) and *state* (non-meditative, meditative). Participants viewed a neutral grating image (contrast = 40%, spatial frequency = 1 cycles/degree) in one eye and an image containing objects to manipulate affordance in the other. To facilitate binocular rivalry, we used images with complementary colours (Wade, 1975), red/black for gratings and green for objects. The green image could contain one of two object pairs (*object-type*)—a teapot with a cup, or a hammer with nail on a slab (Fig. 1). These objects could be in one of two configurations with respect to each other, high-affordance or low-affordance^16^. For high-affordance, it seemed that the teapot was pouring tea into the cup and the hammer was hitting the nail, while for low-affordance the seeming action was not taking place. As all but two participants were right-handed, in the high-affordance configuration, the “acting” objects (hammer and teapot) were in a configuration to be grasped by the right hand. The images were vertically flipped for the two left-handed individuals. Participants were asked to report, using one of the three buttons on a keypad, when their perception switched to the green image, the red image, or a mixture of the two images. In order to maintain consistency in response criteria across individuals, participants were instructed to report the green or red image, when either covered > 80% of the visual aperture, and mixed otherwise.

During the experimental session, each participant underwent two 16-run blocks of the task—one with “non-meditative” instructions, and a second with “meditative” instructions (see below). The order of these task instructions was counterbalanced across participants in the two groups, i.e., within each group, one half of the participants did the non-meditative block first and the meditative block second, while the other half did the opposite. Each 16-run block contained an equal number of object-pair (teapot-cup and hammer-nail) images and affordance (high or low) images. The level of affordance was randomised across runs. The green (object) and red (neutral grating) images were counterbalanced across eyes.

Before starting the meditative block and at the halfway point of the block (i.e., before starting run 9), participants were given 7 min to meditate using the OM technique. The LTMs were familiar with this technique as it was part of their regular practice, while the NVMs were introduced to this technique at the time of recruitment^32^. Before the non-meditative block and at the halfway point, participants did two 3.5-min periods of rest with eyes closed and open respectively, where we measured their baseline EEG activity.

#### Instructions

Some participants were fluent in French while others in English. Before beginning the session, we asked them which of the two languages they would prefer the instructions in, and administered the instructions accordingly. The English instructions were as follows:

Meditative task blocks: “*Rest your mind naturally on the contents of the current image, without controlling, blocking, or grasping what appears. Recognise that what appears on the screen is a mere display of the mind, arising and disappearing spontaneously. Continue following this instruction while reporting the changes in colours, and trying to keep the images fused.*”

Non-meditative task blocks: “*Perform the task as you did during the pre-experiment practice without any specific instructions.*”

#### Self-reports

After each 80-s run, participants were asked to fill out four state questions on a scale of 0–10 about the previous run:

1. How real did you feel the objects to be during the task?
2. How effortless or effortful did it feel to do the task?
3. How calm or stable did you feel your mind to be during the task?
4. How sleepy or awake did you feel during the task?

Here, we were primarily concerned with their responses on the first question (see Supplementary Fig. 5 for responses on the remaining questions), which sought to assess the feeling of subjective reality with respect to the objects inside the green image. In this regard, participants were additionally instructed on how to report subjective reality. We first showed them an image of a chocolate cake and then described that the automatic bodily responses (e.g., salivation) are an outcome of experiencing the image of the cake as something “subjectively real.” In a similar manner we asked them to report how much the objects pairs of hammer/nail and teapot/cup evoked feelings of subjective reality (like hammer hitting the nail, or teapot pouring liquid into the cup) in them for the recently concluded block.

### Binocular rivalry behavioural analysis

Based on the perceptual transition reported by the participants, we calculated the durations for the following three types of percepts: object image (green), neutral image (red), and mixed. Mixed percepts are either brief (e.g., < 1 s), where one dominant image rapidly transitions into the other, or prolonged, where the mixed state persists for several seconds. If the participants reported a mixed percept that was less than 1 s, we took it as a perceptual transition and assigned half of its duration to the percept reported before, and the half to the one reported after.

We then averaged the mean duration for each of the three types of percepts (green, red, mixed) within each run. To behaviourally quantify affordance, we calculated the ratio of mean durations of the green (object) image to the red (neutral) image separately for the high-affordance (bHAR) and low-affordance (bLAR) images and for the two image-types (hammer/nail, teapot/cup) again on a run-by-run basis (because the duration ratio was positive definite with long tails, we instead used log of the duration ratio as our dependent variable for statistical analyses), while excluding any mixed percepts. This ratio served as a behavioural index of affordance.

### Meditation practices

OM practices aim to cultivate and sustain an effortless, open and accepting awareness of present moment experience, without being reactive or absorbed in its contents^8,34^. This cognitive perspective allows one to recognize that sensations, emotions, and thoughts are simply mental events, and thus do not necessarily need to be acted upon. A more advanced form of OM is “Open Presence” (OP) which is a paradigmatic case of a so-called non-dual mindfulness meditation in Tibetan Buddhist traditions^34^. Styles of meditation that cultivate OP are described as inducing a phenomenal experience where the intentional structure Involving the duality between object and subject is attenuated^9,34^. Open Presence (OP) style of practices can be found in both the Dzogchen and Mahamudra traditions of Tibetan Buddhist meditation, that have as central tenet the cultivation of the OP state. An example instruction goes as follows: “Within a state free of hopes and fears, devoid of evaluation or judgement, be carefree and open. And within that state, do not linger on the past; do not invite the future; place awareness within the present, without alteration, without hopes or fears”^34^. Based on its traditional presentation^35^, OP practice is considered here an advanced form of OM practice. Theoretically, OP meditation consists of a state where the phenomenological qualities of effortlessness, openness and dereification are vividly experienced and control-oriented elaborative processes are reduced to a minimum. Getting familiar and stabilising the suspension of these elaborative processes requires substantial training for instance during a traditional 3-year meditation retreat as the one done by the experts of this study. For this reason, the term OP is used here only for expert meditators, even if both experts and novices received the same meditation instructions during the task. Below we use the term OM for both novices and experts and we will assume that for experts only it could also qualify as a practice of OP.

### EEG acquisition

EEG data were acquired using a 64-channel Biosemi system at a sampling frequency of 512 Hz in the standard 10/20 configuration in addition to mastoids. Each channel was adjusted to have impedance below 18 kΩ before beginning the experiment.

### Missing data and excluded participants

One participant was excluded from EEG analysis due to technical difficulties during EEG acquisition.

Two LTMs and one NVM reported not seeing grating images for one of the four conditions (2 blocks × 2 affordance) at all, which precluded calculating our dependent variable (ratio of object to grating images). Two LTMs and one NVM had highly disproportional eye dominance and did not report seeing the image in one eye for one of the four conditions. We excluded these 6 participants from behavioural and EEG analysis (see Table 1 for demographics of the final sample).

**Table 1:** Participant demographics.

	n	Sex	Age	Education
Novices	20	9F/11M	53.9 (7.6)	4.0 (2.2)
LTMs	14	4F/10M	53.3 (7.1)	2.7 (2.5)
*p*		0.54	0.83	0.12

The questionnaire data could not be obtained on 8 of the 40 participants (6 LTMs and 2 NVMs).

### Preprocessing

EEG preprocessing was conducted using EEGLAB^36^ and custom MATLAB code. We first downsampled the raw EEG to 256 Hz and band-pass filtered between 0.1 and 80 Hz as well as notch filtered with a 1 Hz bandwidth to exclude electrical line noise at 50 Hz. After keeping only the rivalry task, rest and meditation blocks, we then visually inspected the data to remove noisy channels. We then re-referenced the data to the average of the mastoids and used Independent Component Analysis (ICA) implemented in EEGLAB (*binica* algorithm) to manually select and remove components reflecting ocular and muscle artefacts (Delorme et al., 2007). Subsequently, we flagged any epochs where voltage at a channel exceeded ± 120 µv within a 200 ms window to exclude from analysis.

#### µ-rhythm

During binocular rivalry participants press a button at the perceptual transitions. We wanted to evaluate the change in µ to image affordances. Moreover, we expected that there might be µ/alpha desynchronisation at perceptual transitions simply due to attentional effects because transients are more dynamic or “interesting” than stable percept periods. We thus calculated the average *µ* power for each run by excluding [− 1, + 0.6] s around each transition. The exclusion window preceding the transition was larger than the one post transition because of the delay between the actual perceptual transition and motor response^37^. To calculate the average *µ* power, we bandpass filtered the EEG data within 8–12 Hz and then took the absolute value of the Hilbert transform using the MATLAB functionffect*t*. *µ* power was further averaged across the channels C1, C3, FC1, FC3, CP1, CP3, for right handers and C2, C4, FC2, FC4, CP2, CP4, for left handers. We also performed exploratory analysis using the alpha rhythm at posterior channels (averaging Oz, O1, O2, pOz, PO3, PO4, PO7, PO8, and Pz). While the association of alpha with binocular rivalry is sometimes measured over a wider frequency range (e.g., 7–13 Hz^38^), we restricted the alpha analysis also to the same frequency range as *µ* (i.e., 8–12 Hz) to allow comparison.

### Statistical analyses

We used linear mixed models implemented in R^39^ via the package *lme4* (Linear Mixed Effects version 4)^40^ for data analyses. Depending on the analysis, one or more independent variables among *Group* (LTM, NVM), *State* (BL, OM), *Affordance-level* (high, low) and *Percept* (green, red, mixed), were entered as fixed effects. *Subject* was systematically included as a random factor, and all main within-subject fixed effects were included as by-subject random slopes. *Run number* (1–32) was included both as a fixed effect and a by-subject random slope to regress out subject-specific temporal effects over the course of the experiment. The inclusion of interaction terms in the structure of random effects caused convergence issues in some models; therefore, and for the sake of consistency, slopes for interaction terms between *Group*, *Affordance-level*, and *Percept* were dropped altogether from the random effects in all models^41^. For behavioural and electrophysiological measures, the response variables were log-transformed to approach a normal distribution; statistical inference was performed using an ANOVA analysis of variance with the Satterthwaite approximation for degrees-of-freedom. For self-report data such as subjective realism, a generalized mixed model with a Poisson distribution was more appropriate, and chi-square tests were used for statistical inference. Paired t-tests, corrected for multiple comparisons using Tukey honestly significant difference test (HSD), were used as post-hoc tests comparing estimated marginal means.

For correlation analyses between behavioural and electrophysiological measures (occipital alpha and alpha *µ* power), we used multilevel partial Spearman correlation models as implemented in the R package *correlations*^42^. This implementation harnesses mixed effect models to estimate intra-individual correlation coefficients at the level of the group. Consistent with other analyses in this study, the variables were log-transformed (although this is not strictly necessary with *Spearman* correlation as it operates on ranks, which are insensitive to monotonic transformations). *Partial* correlations allowed to reduce potential confounding effects due to crosstalk between central and occipital electrodes.

## Results

### Hypothesised results

#### Behavioural affordance ratio changes with mindfulness and between groups

**Figure 2:**
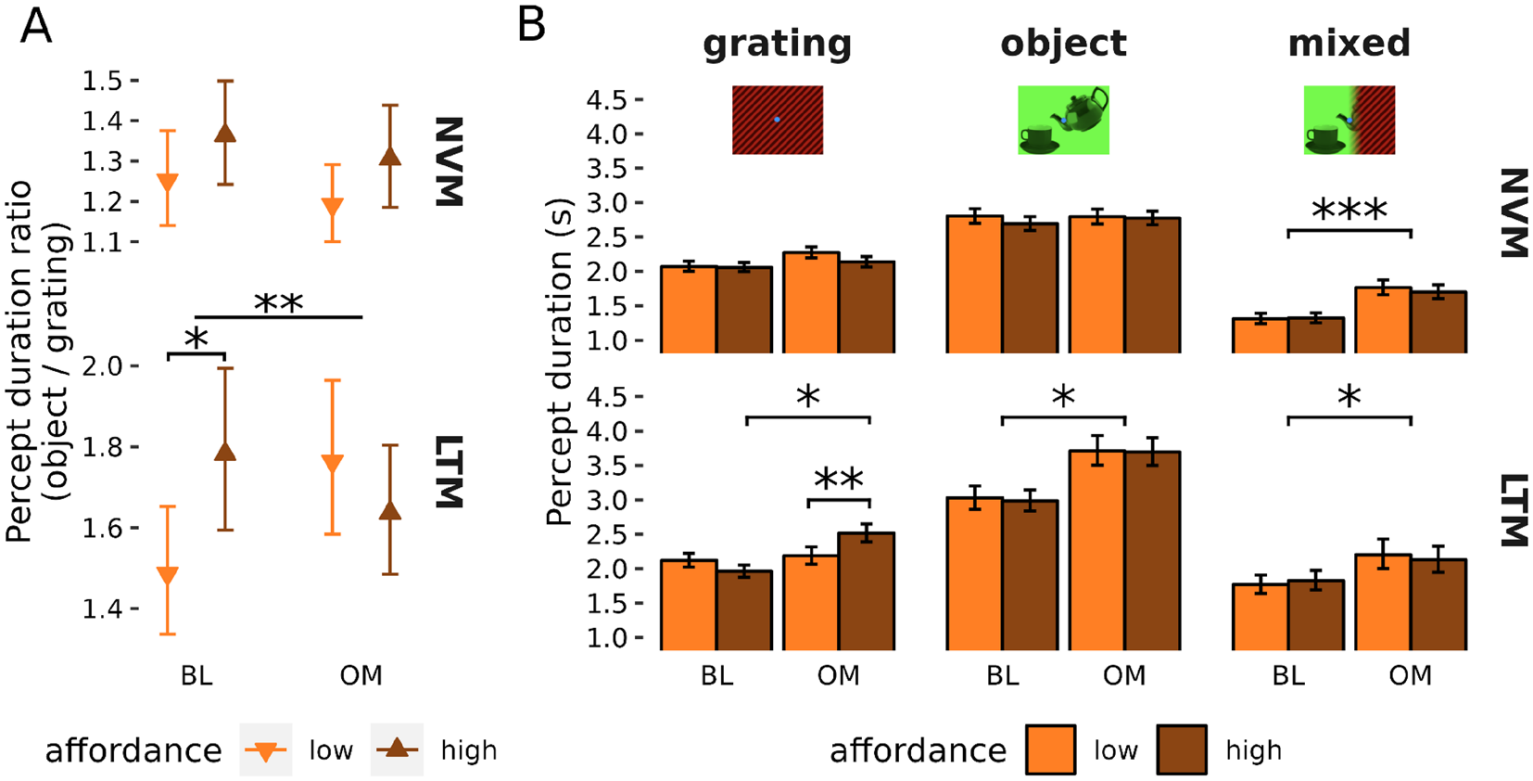
(**A**) The ratios of dominant percept durations for object image to grating image (while excluding any mixed percepts) for the two groups of participants, novices (NVM) and long-term meditators (LTM) for the two states BL and OM and the two levels of affordance (low and high). (**B**) Dominant percept durations for the two groups, two states, two affordance levels and three types of percepts: grating, object and mixed. Error bars are within-subject standard errors of the means. *p < 0.05, **p < 0.01, ***p < 0.001.

Figure 2 shows the behavioural results. Our primary behavioural dependent variable was the ratio of mean percept duration of the object image (teapot-cup and hammer-nail) to mean percept duration of the neutral image (grating) for each run separately, and this could be in the high- or low-affordance configurations (bHAR and bLAR, respectively). For the affordance effect to have been successfully manipulated behaviourally, we hypothesised bHAR > bLAR when combined across all subjects. There was a trend for this effect (bHAR/bLAR = 1.12, 95% CI [0.99, 1.26], *t*(25.7) = 1.93, *p* = 0.067; Fig. 2A). We also hypothesised that the two groups would exhibit a different change in the affordance effect for OM versus BL. Specifically, the affordance effect (i.e., bHAR > bLAR) would be reduced in the LTMs following OM, but not in the NOVs. This would show up statistically as a 3-way interaction between *State*, *Affordance-level* and *Group*. There was indeed a significant 3-way interaction (*F*(1, 903) = 5.06, *p* = 0.025). Within the LTMs, there was a significant double-difference between *State* and *Affordance-level* (*t*(892) = 2.84, *p* = 0.005), with bHAR > bLAR during BL (bHAR/bLAR = 1.20, 95% CI [1.01, 1.41], *t*(41.6) = 2.17, *p* = 0.036) but not following OM (bHAR/bLAR = 0.93, 95% CI [0.78, 1.09], *t*(43.8) = − 0.93, *p* = 0.36). There was no such double difference in the NOVs (*t*(915) = 0.19, *p* = 0.85).

#### No change in affordance for µ-rhythm

Next, we hypothesised that *µ* power for runs with high-affordance images (mHA) would be lower than low-affordance images (mLA) during BL across the groups. To perform this analysis, we modelled *µ* log-power at the level of individual percepts while excluding the period around perceptual transitions (see Methods) and ignoring percept types. Thereafter we report effects as ratios of untransformed power.

**Figure 3:**
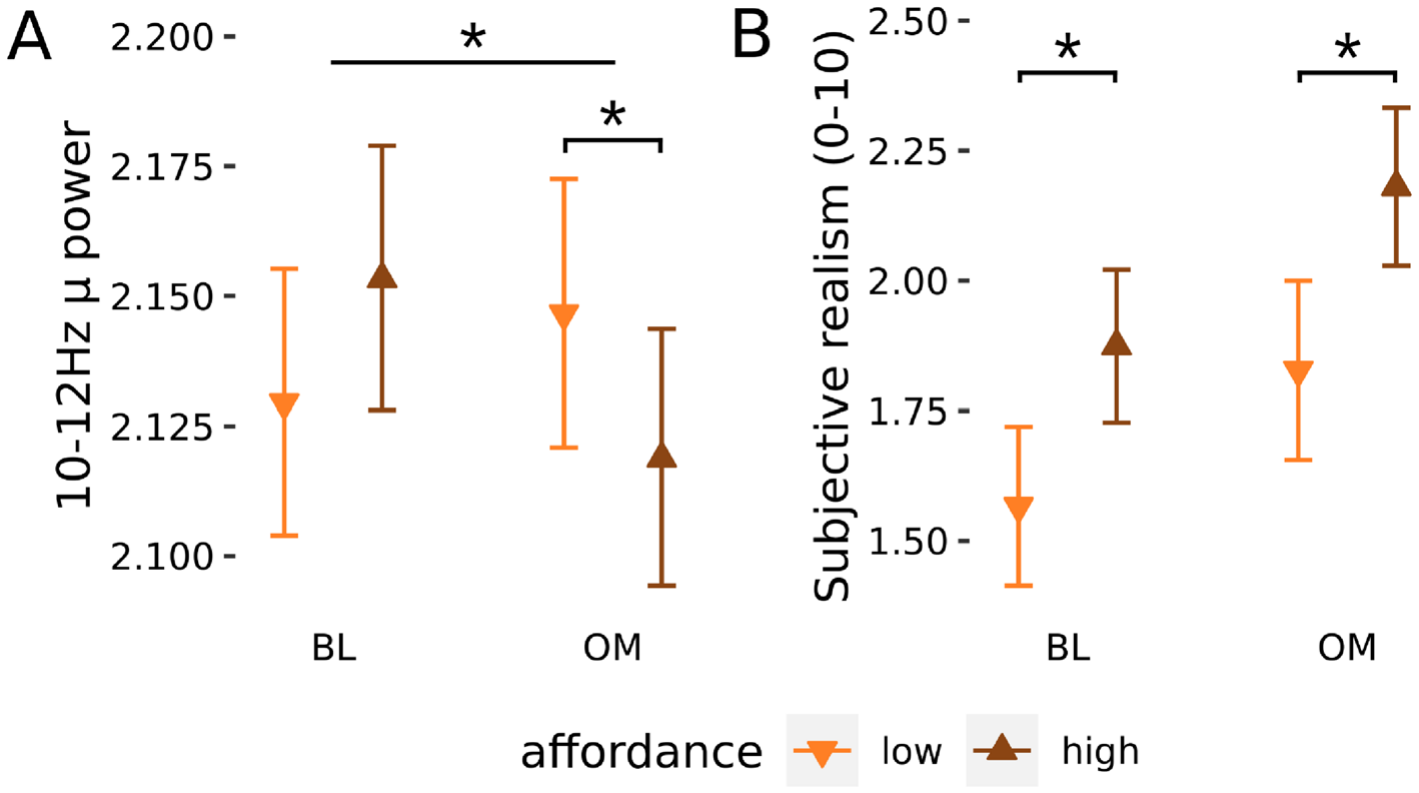
(**A**) Power of upper µ rhythm (10–12 Hz) for the two affordance-levels and two states averaged across the two groups due to the lack of a 3-way interaction. (**B**) Self-reported “subjective realism” of the object image for the two affordance-levels and two states also averaged across the two groups. Error bars are within-subject standard errors of the means. *p < 0.05.

The mHA/mLA ratio during BL was not statistically significant (F(1,35.0) = 1.41, *p* = 0.24). There was also no 3-way interaction between *State*, *Affordance-level* and *Group* (F(1,16,425) = 2.97; *p* = 0.085). We did however find an unexpected 2-way interaction between *State* and *Affordance-level* (F(1,16,190) = 7.41; *p* = 0.006) (Fig. 3A). Contrary to our hypothesis, μ power was not different for high-affordance compared to low-affordance during BL (mHA/mLA = 1.01, 95% CI [0.99, 1.03], t(72.9) = 1.28; *p* = 0.20), however, and in support of our prediction, it was slightly lower for high-affordance compared to low-affordance during OM (mHA/mLA = 0.98, 95% CI [0.96, 1.00], t(80.7) = –2.20; *p* = 0.031). In other words, there was *µ* desynchronisation for high-affordance images during OM and not BL; the expected direction for an affordance effect but with a state specificity opposite of what we hypothesized.

These surprising results may reflect an unexpected effect of spill over of alpha rather than *µ* desynchronisation, where the more interesting high-affordance stimulus drew greater visual attention on part of the participants, and especially so during OM when they cultivated a heightened attention. We tested this possibility by modifying the statistical model to control for alpha power, but the pattern of results did not change (Supplementary table 1), suggesting a genuineffectct of *µ* desynchronisation. Another possibility is that the phenomenon of affordance-related neural desynchronisation may have waned over the course of the experiment—as suggested by the saturation of *µ* power in the second half of the experiment (Supplementary Fig. 1)—thus resulting in attenuated effect sizes. Interestingly, restricting the statistical analysis to the first half of the experiment, before µ power saturates at high values, supports this idea as it indicates that the effect of meditative state on affordance is specific to LTMs (Supplementary Table 2), a specificity that was already visible on the patterns of means calculated from the whole dataset (Supplementary Fig. 2).

#### Higher subjectively experienced realism for high vs. low affordance

We also hypothesised that reports of subjective realism would be higher for high-affordance versus low-affordance during baseline. We found such an effect across the groups (sHAR/sLAR = 1.18, 95% CI [1.02, 1.38], *z* = 2.19, *p* = 0.029; Fig. 3B). Contrary to our prediction, there was no significant 3-way interaction between *State*, *Affordance-level* and *Group* (χ^2^(1) = 0.41; *p* = 0.52) for subjective realism or any significant 2-way interactions (all *p* > 0.81, see also the distribution of data by condition in supplementary Fig. 4).

### Exploratory results

#### Evolutions over the course of the experiment

All our statistical models included a regressor for Run to take into account potential temporal effects over the course of the experiment. Although we had no prior hypothesis regarding such effects, we report them here for completeness.

The power of the μ-rhythm increased significantly between the first and the last runs of the experiment (first run = 1.97, 95% CI [1.62, 2.41]; last run = 2.31, [1.86, 2.88]; t(28.2) = 5.39, *p* < 0.0001), which is consistent with systematic increase in alpha power over task period found in previous work^43^.

The behavioural affordance ratio (i.e. the ratio of mean percept duration of the object image to mean percept duration of the neutral, grating image) increased significantly over the course of the experiment (first run = 1.31, 95% CI [1.13, 1.53]; last run = 1.62, [1.40, 1.86]; t(27.5) = 2.82, p = 0.009). Previous work has shown that rivalry alternations slow down over the course of an experiment^44^. Our finding suggests moreover that more high-level stimuli like objects may get prolonged in duration more compared to low-level grating stimuli.

#### Prolonged percept durations following meditation

Our main hypothesis for the behavioural affordance effect was based on duration ratios of the object to the grating image. However, past research has also shown that binocular rivalry^45^ and other perceptual rivalry^46,47^ durations can generally be prolonged through meditation with our recent study pointing specifically towards prolonged mixed percepts in binocular rivalry^48^. We thus explored the effects of *State*, *Affordance-level, Group* and *Percept type* (object, grating, mixed), on the binocular rivalry duration data (Fig. 2B). A linear mixed model of the data revealed a significant 4-way interaction between these factors (F(2,23,848) = 7.08, *p* < 0.001).

To better understand this complex interaction, we modelled *State*, *Affordance-level,* and *Percept* in the two groups separately. In NVMs, there was no significant 3-way interaction for this model (F(2,15,082) = 1.16; *p* = 0.31) but a significant 2-way interaction between *State* and *Percept* (F(2, 14,728) = 39.46, *p* < 0.0001). Post-hoc analysis revealed that this interaction was due to an increase in mixed percept durations for OM versus BL (OM/BL = 1.28, 95% CI [1.17, 1.41]; t(25.5) = 5.40; *p* < 0.001), with no such difference present for object images (OM/BL = 0.99, 95% CI [0.90, 1.08]; t(20.1) = -0.26; *p* = 0.80) nor gratings (OM/BL = 1.08, 95% CI [0.98, 1.18]; t(20.5) = 1.72; *p* = 0.10).

For the LTMs, there was a significant 3-way interaction between *State*, *Affordance-level*, and *Percept* (F(2,8801) = 6.77, *p* = 0.001). Further analysis for each percept type revealed that this interaction was driven by a double difference between *State* and *Affordance-level* for the grating percept (t(8777) = 3.59, *p* < 0.001), but not for the object (t(8743) = − 1.20, *p* = 0.23) nor mixed percepts (t(8784) = − 0.58, *p* = 0.57). This double difference for the grating images was driven by longer durations during OM compared to BL for high-affordance (OMHA/BLHA = 1.18, 95% CI [1.03, 1.34], t(22.8) = 2.58, *p* = 0.017) but not for low-affordance conditions (OMLA/BLLA = 0.97, 95% CI [0.85, 1.11], t(22.8) = -0.44, *p* = 0.67). Similarly, durations of gratings were longer for high-affordance compared to low-affordance in OM (OMHA/OMLA = 1.12, 95% CI [1.04, 1.21], t(8663) = 3.00, *p* = 0.003) while the opposite was observed in BL (BLHA/BLLA = 0.93, 95% CI [0.87, 1.00], t(8804) =  − 2.04, *p* = 0.04).

In contrast to the pattern found in novice practitioners, who reported longer durations in OM only for mixed percepts, LTMs showed an increased persistence of all percepts (OM/BL = 1.13, 95% CI [1.01, 1.27]; t(12.7) = 2.31; *p* = 0.038) with the exception of gratings during low affordance runs.

#### Association between percept durations, alpha and µ-rhythm

The EEG hypothesis of the present study was based on the premise that *µ* rhythm is functionally distinct from visual alpha in that it is related to motor preparation and we would observe its effects according to manipulations of affordance-induced motor preparation in our stimuli. Our experimental design allowed us to test an exploratory hypothesis where *µ* and alpha would be differently related to trial-by-trial fluctuations of bistable percept durations. Specifically, recent work has shown that trial-by-trial fluctuations in bistable perceptual durations using a Necker cube stimulus are positively correlated with parieto-occipital (visual) alpha power^49,50^. However, if *µ* rhythm is specifically related to affordance differences between the two concurrently presented images (and is different from visual alpha), we would expect a *negative* correlation of µ power with durations of object percepts and not grating percepts. We performed this exploratory analysis to test whether trial-by-trial fluctuations in parieto-occipital alpha and sensorimotor µ predicted percept durations during our binocular rivalry task. We performed this analysis specifically on dominant percepts (i.e., excluding mixed percepts).

**Figure 4:**
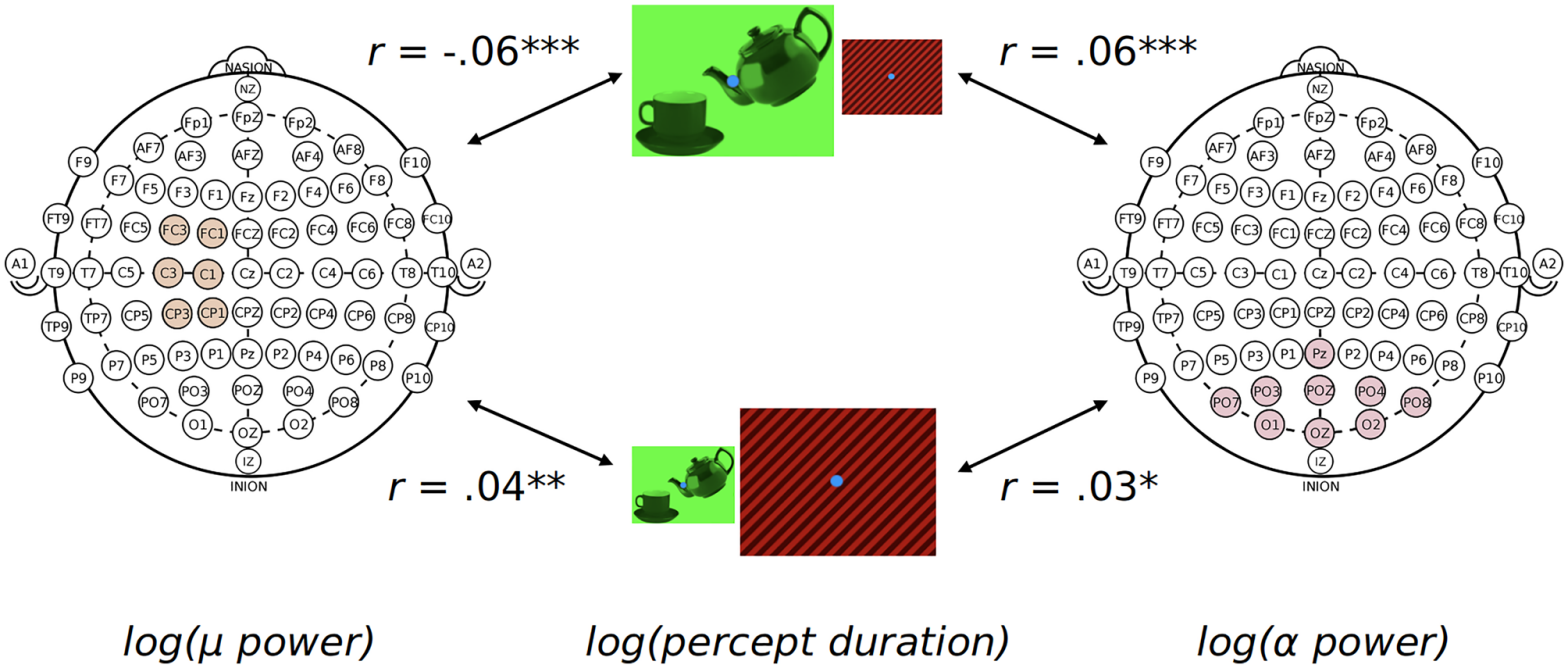
Multilevel correlation between percept duration and somatomotor *µ*-rhythm power (left) and occipital alpha power. While grating images were positively correlated with both alpha and µ (below), object image was positively correlated with alpha but negatively correlated with µ. *p < .05, **p < .01, ***p < .0001.

At a trial level, there was a significant positive multilevel correlation between parieto-occipital alpha and percept duration separately both for the object image (ρ(7439) = 0.060, 95% CI [0.036, 0.083]; *p* < 0.0001) and the grating image (ρ(6078) = 0.032, 95% CI [0.006, 0.057]; p = 0.014) (Fig. 4, right), replicating previous work^49,50^. Interestingly, there was a highly significant negative multilevel correlation between *µ* and percept duration of the object image (ρ(7439) = –0.057, 95% CI [–0.081, –0.034]; p < 0.0001). This was despite a positive multilevel correlation between µ and percept duration of the grating image (*ρ*(6078) = 0.044, 95% CI [0.018, 0.070]; *p* = 0.001) (Fig. 4 left, all reported p-values are raw but survive correction for multiple comparison).

## Discussion

Contemplative traditional literature has long suggested that long-term practice of meditation may enable cultivation of unique phenomenological states and prominently one where meditators can cultivate a radical meta-perspective towards mental activity where it appears within consciousness simply as “ongoing phenomena”^4,7,9^. This has been labelled among other things as de-reification, or the process of not reifying mental contents (i.e., not experiencing them as something having a fully objective reality)^4^. Open monitoring (OM) meditation—and in more advanced meditators, open presence (OP) meditation—has been proposed to enable such a phenomenological outlook. Here, we tested if long-term meditators (LTM) could de-reify from visual stimuli that would otherwise automatically afford actions by cultivating a de-reified stance following a period of OP practice in a way that novice meditators (NVM) might not be able to after OM practice. Participants belonging to an LTM and a NVM group were asked to try to maintain the de-reified phenomenological stance cultivated during the meditation period, while we measured their behaviour, neural correlates and self-reports in comparison to a baseline state where they were asked to not cultivate a de-reified stance.

Our primary hypothesis, that the ratio of binocular rivalry durations of high-affordance-to-neutral images would be greater than the ratio of low-affordance-to-neutral image durations (i.e., the behavioural affordance effect) during the baseline condition across both groups, was weakly supported with a trend in the expected direction. We did however find a significant effect for our second (and key) behavioural hypothesis—the two groups exhibited the behavioural affordance effect differently in the meditative vs. baseline states. When specifically considering LTMs, there was a significant behavioural affordance effect at baseline, with such an effect abolished for the meditative state, as hypothesised. For the NVMs the behavioural affordance effect, while not statistically significant, and remained as a weak trend both during baseline and for the meditative state. Overall, these results support the idea that the long-term meditators were able to cultivate a state where they perceived their mental objects as “ongoing mental phenomena,” i.e., in their raw sensory existence with the absence of supplementary cognitive processing that would compel individuals to take physical or mental action upon their perception^4,9^. Our results suggest that such suspension of supplementary cognitive processing may happen even for very basic level action planning that is otherwise considered to be an automatic process. Further studies with less advanced practitioners could test whether this behavioural effect is also an outcome of OM meditation, in addition to OP meditation, and if it is also an outcome of other (e.g., concentrative) meditation practices that have previously been related to altered binocular rivalry dynamics^45,48^.

Past research has shown that mental processing of action affordance can be mitigated to some extent by diverting attention away from action-related attributes of objects^18,20^. While such an explanation cannot be entirely ruled out in relation to findings, our observation that mitigation of affordance processing was specific to long-term meditators (and not novices) suggests that it was likely not simply due to diverted attention effects, which if anything would be more in the group of novices who have not engaged in prior attention training through meditation.

Our primary behavioural manipulation of the affordance effect was relatively weak. Past studies have found preferential processing of implied action between pairs of stimuli of the kind we used in the present study through multiple paradigms^15^. In their classic study, Riddoch et al.^16^ found that neuropsychological patients with visual extinction—a phenomenon where patients with lesions fail to note stimuli contralateral to the lesion when presented with stimuli on the ipsilateral side—exhibited less extinction when stimuli afforded action compared to when they did not. Subsequently, other studies with non-clinical patients have also shown that when objects affording actions are presented simultaneously, they lead to better semantic processing^21^, visual discrimination of the objects^17^, and greater BOLD activity in object and shape selective cortex (lateral occipital cortex and anterior fusiform cortex)^51^. Previous studies had not tested if preferential processing of images that afford actions can be observed using directions of dominant percepts during binocular rivalry. A few studies have however shown that actual physical actions corresponding to one of the bistable visual percepts do increase the dominance duration of that percept for binocular rivalry^22,23^ and other forms of perceptual rivalry^52^. Our observation of a weaker effect of afforded actions on binocular rivalry may have been due to a generally weaker modulatory effect of afforded versus actual physical actions, or because of the lack of saliency of affordance using affective contents (e.g., appetitive content^3^). Another possible reason for the effect to be weak may have been the use of only two different stimuli that may have rapidly become uninteresting to the subjects. Interestingly, we found that during baseline the affordance effect was stronger in the LTM group compared to the NVM group. This could have been due to the LTMs generally paying more attention to the task (for example, due to greater motivation).

Besides our main behavioural hypotheses comparing afforded actions, we also found that during the meditative state participants from both groups reported an increase in mixed percepts compared to baseline. This replicates recent work also showing an increase in mixed percepts following meditation compared to baseline, though only in the LTM group^48^. The two studies together suggest that meditation may be generally associated with increased mixed percepts. Current computational models of rivalry suggest that increased mixed percepts may arise due to two potential mechanisms, (1) reduced excitation/inhibition in early visual areas^53,54^, or (2) in the process of perceptual inference model^55^ where priors for interpreting the sensory stimulus as being in one of two bistable states are relaxed. A more thorough comparison of these accounts in future work may offer mechanistic insight into the neurobiological correlates of a de-reified phenomenological attitude cultivated through meditation.

We also hypothesised that the µ power (across participants) during high-affordance runs would be smaller during baseline compared to low-affordance runs (i.e., neural affordance effect) and that this effect would be abolished in the LTMs during the meditative state. We did not find this to be the case. Instead, we found the neural affordance effect across both groups for the meditative state, with no such difference during baseline. Past studies have shown that action preparation, including in response to viewing images that afford actions, can lead to desynchronisation of the *µ* rhythm^24^. One reason we may not have observed *µ* desynchronisation for the high- compared to low-affordance configuration of the stimuli is due to the constant motor activity that is needed for reporting perception during a continuous binocular rivalry task irrespective of the affordance configuration of the stimuli. Despite excluding periods around reported perceptual transitions to prevent motor contamination of µ rhythm during analysis, it is possible that we were not able to fully avoid it. This is because binocular rivalry stimuli can appear to be very perceptually dynamic with repeated occasions where it seems that the percept is about to switch but it does not. At such moments, one may expect that the participants would be ready with a response, but may not ultimately press the button. Such periods of motor activity would add noise to the µ rhythm, and prevent us from measuring its desynchronisation in relation to the affordance manipulation.

On the other hand, we did find that the pattern of affordance-related* µ* desynchronisation differed between states, although post-hoc tests indicate that it is unexpectedly driven by an affordance effect in the meditative state rather than in the baseline. Additional analyses ruled out the possibility that these results reflect an effect of spill over of alpha rather than *µ* desynchronisation. Future studies should be optimized and better powered to address the question of how meditation states and expertise affect affordance-related* µ* desynchronisation.

Finally, Likert ratings revealed that across participants and states (i.e., baseline vs. post-meditative), individuals found the high-affordance configuration of the images to be more subjectively real than the low-affordance images confirming the validity of our manipulation, irrespective of group or state. While this sample size may have been sufficient to test for a main effect of affordance, the statistical power for more complex interactions was probably too low.

Besides our main hypotheses, our results replicated recent work showing a positive correlation between binocular rivalry dominance durations and occipital alpha power^49,50^. Here, alpha, being an inhibitory rhythm, may reflect the depth of suppression of the non-dominant percept during rivalry, which would in turn be related to a general increase in dominance durations. Interestingly, we found that µ rhythm was *negatively* correlated with duration of the object images and *positively* with the duration of grating images. This is consistent with the idea that increased neural processing of action preparation as indexed by *µ* desynchronization is associated with higher perceptual dominance of object images, i.e., longer object percepts and shorter grating percepts. These results show for the first time, to our knowledge, that alpha and µ rhythms can separately characterise the contribution of visual and motor preparatory aspects of a visual stimulus in visual perception, and more generally extend the evidence for association between bistable perception and the alpha rhythm^38,49^. Future studies could explore the relationship between alpha and µ activities and the changes across various phenomenological dimensions of dereification (e.g., sense of grasping, meta-awareness, degree of subjective realism, see ^10^) or how alpha and µ are related to experiential changes in meditation^56^.

Overall, while our behavioural results support the key idea behind this study—that open monitoring meditation can prevent automatic mental action processing of images that afford actions in LTMs—the lack of a statistically significant effect in NVMs limit the generalisability of our finding. We also observed µ desynchronization positively correlated with percept durations despite occipital alpha having a negative correlation indicating its potential for use as a signature of affordance in rivalry paradigms, though it was not sensitive to experimental manipulation. We recommend that future studies revisit this question using a task which has previously been established to show an effect of action preparation both behaviourally and neurally.

### Supplementary Information

Supplementary Information.

## Data Availability

The code and preprocessed data that support the findings of this study are openly available at https://osf.io/njycg/.
