# Supplementary material for: Reduced processing of afforded actions while observing mental content as ongoing mental phenomena
Source: Sci Rep. 2024 May 2;14:10130. doi: 10.1038/s41598-024-60934-6 (PMC11065984; doi:10.1038/s41598-024-60934-6)
Supplement: Supplementary file 1 — Supplementary Information. [file 41598_2024_60934_MOESM1_ESM.docx]

**Reduced processing of afforded actions while observing mental content as ongoing mental phenomena**

Sucharit Katyal^1,2^, Oussama Abdoun^1^, Hugues Mounier^3^, Antoine Lutz^1^

# Supplementary Materials

#

# Supplementary Tables

|  | **object/grating duration ratio** | |
| --- | --- | --- |
| *Predictors* | *Estimate (95%CI)* | *p* |
| Group [LTM / NVM] | 0.877  (0.776 – 0.992) | **0.037** |
| State [OM / BL] | 0.986  (0.949 – 1.024) | 0.465 |
| Affordance [high / low] | 0.972  (0.928 – 1.017) | 0.214 |
| Run [n+1 / n] | 1.007  (1.002 – 1.011) | **0.005** |
| Group x State | 1.027  (0.990 – 1.066) | 0.154 |
| Group x Affordance | 0.996  (0.953 – 1.042) | 0.871 |
| State x Affordance | 0.970  (0.942 – 0.999) | **0.046** |
| Group x State x Affordance | 1.034  (1.004 – 1.065) | **0.025** |
| **Random Effects** | | |
| σ^2^ | 0.218 | |
| τ_00_ _subject: intercept_ | 0.158 | |
| τ_11_ _subject: state_ | 0.000 | |
| τ_11_ _subject: affordance_ | 0.010 | |
| τ_11_ _subject: run_ | 0.000 | |
| ρ_01_ | 0.659 | |
|  | 0.017 | |
|  | -0.501 | |
| ICC | 0.388 | |
| N _id_ | 33 | |
| Observations | 995 | |
| Marginal R^2^ / Conditional R^2^ | 0.062 / 0.426 | |

**Supplementary table 1.** Estimates for the model of the log-ratio of object/grating durations. Fixed parameters have been exponentiated. σ^2^ is the residual variance of the model, τ are by-subject random variances and ρ_01_ are correlations between the random intercept and random slopes.

|  | **full dataset** | | **with alpha power covariate** | | **1st half of experiment** | | **2nd half of experiment** | |
| --- | --- | --- | --- | --- | --- | --- | --- | --- |
| *Predictors* | *Estimate (95%CI)* | *p* | *Estimate (95%CI)* | *p* | *Estimate (95%CI)* | *p* | *Estimate (95%CI)* | *p* |
| Group [LTM / NVM] | 0.984  (0.675 – 1.435) | 0.933 | 0.892  (0.674 – 1.182) | 0.426 | 0.881  (0.692 – 1.122) | 0.305 | 0.822  (0.642 – 1.051) | 0.118 |
| State [OM / BL] | 1.006  (0.978 – 1.034) | 0.697 | 1.003  (0.986 – 1.019) | 0.764 | 0.995  (0.762 – 1.298) | 0.969 | 0.968  (0.742 – 1.264) | 0.813 |
| Affordance [high / low] | 0.995  (0.980 – 1.011) | 0.555 | 0.994  (0.981 – 1.008) | 0.400 | 0.995  (0.980 – 1.011) | 0.552 | 0.998  (0.979 – 1.017) | 0.846 |
| Run [n+1 / n] | 1.005  (1.003 – 1.007) | **<0.001** | 1.003  (1.001 – 1.004) | **<0.001** | 1.006  (1.002 – 1.009) | **<0.001** | 1.001  (0.999 – 1.003) | 0.377 |
| Group x State | 0.974  (0.924 – 1.027) | 0.336 | 0.986  (0.954 – 1.019) | 0.405 | 0.995  (0.613 – 1.613) | 0.983 | 1.035  (0.625 – 1.714) | 0.893 |
| Group x Affordance | 0.988  (0.959 – 1.019) | 0.444 | 0.979  (0.954 – 1.005) | 0.117 | 0.980  (0.950 – 1.011) | 0.197 | 0.985  (0.948 – 1.022) | 0.420 |
| State x Affordance | 0.967  (0.947 – 0.988) | **0.002** | 0.978  (0.959 – 0.997) | **0.021** | 0.991  (0.961 – 1.023) | 0.588 | 0.982  (0.946 – 1.019) | 0.337 |
| Group x State x Affordance | 0.963  (0.923 – 1.005) | 0.085 | 0.967  (0.930 – 1.005) | 0.084 | 0.939  (0.882 – 0.999) | **0.047** | 1.007  (0.934 – 1.086) | 0.851 |
| log(alpha power) |  |  | 1.486  (1.397 – 1.581) | **<0.001** | 1.437  (1.349 – 1.531) | **<0.001** | 1.525  (1.435 – 1.622) | **<0.001** |
| **Random Effects** | | | | | | | | |
| σ^2^ | 0.114 | | 0.094 | | 0.093 | | 0.095 | |
| τ_00 subject: intercept_ | 0.307 | | 0.168 | | 0.065 | | 0.074 | |
| τ_11 subject: state_ | 0.001 | | 0.000 | | 0.097 | | 0.125 | |
| τ_11 subject: affordance_ | 0.000 | | 0.000 | | 0.000 | | 0.000 | |
| τ_11 subject: run_ | 0.000 | | 0.000 | | 0.000 | | 0.000 | |
| τ_11 subject: log(alpha)_ |  | | 0.030 | | 0.030 | | 0.027 | |
| ρ_01_ | -0.066 | | -0.307 | | -0.404 | | 0.686 | |
|  | 0.041 | | 0.140 | | 0.265 | | 0.352 | |
|  | 0.257 | | 0.095 | | 0.013 | | -0.751 | |
|  |  | | 0.202 | | 0.319 | | 0.497 | |
| ICC | 0.748 | | 0.708 | | 0.604 | | 0.548 | |
| N | 33 _id_ | | 33 _id_ | | 33 _id_ | | 33 _id_ | |
| Observations | 17067 | | 17067 | | 8580 | | 8487 | |
| Marginal R^2^ / Conditional R^2^ | 0.005 / 0.749 | | 0.163 / 0.755 | | 0.176 / 0.674 | | 0.285 / 0.677 | |

**Supplementary table 2.** Regression estimates for planned and exploratory models of log(µ power). Fixed parameters have been exponentiated. σ^2^ is the residual variance of the model, τ are by-subject random variances and ρ_01_ are correlations between the random intercept and random slopes. Although regression coefficients and the marginal R2 suggest that alpha power predicts most µ power variability, effects of experimental manipulations remain significant even when alpha power is included as a covariate (compare second and first column). Such effects were mostly detected during the first part of the experiment (third column), before alpha and µ power saturated at high levels (see supplementary figure 1).

# Supplementary Figures


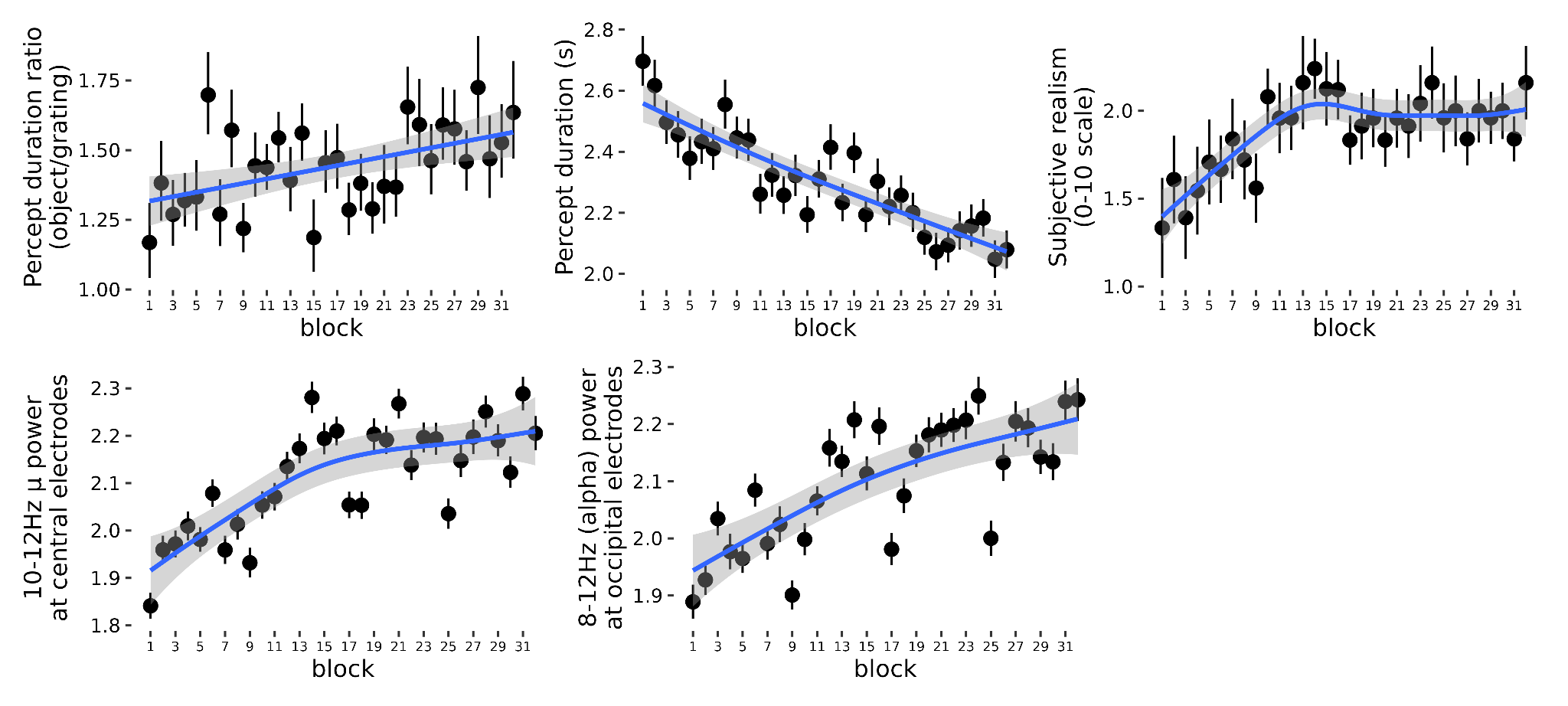


**Supplementary figure 1.** Temporal evolution of behavioral, neural and self-reported measures over the course of the experiment. Smooth lines are the result of thin plate spline regression over the entire dataset. Error bars are within-subject standard errors of the means.


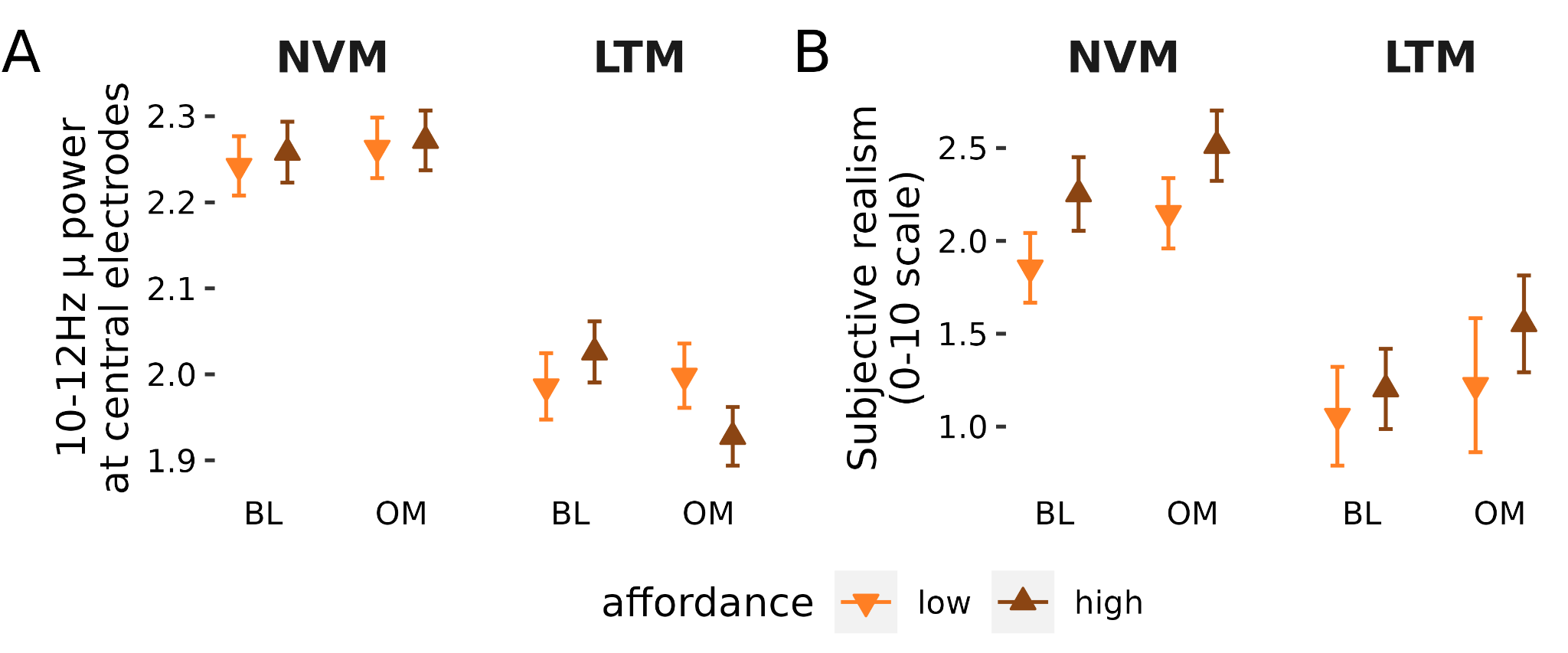


**Supplementary figure 2.** Effect of experimental manipulations on central *µ* power. Error bars are within-subject standard errors of the means.


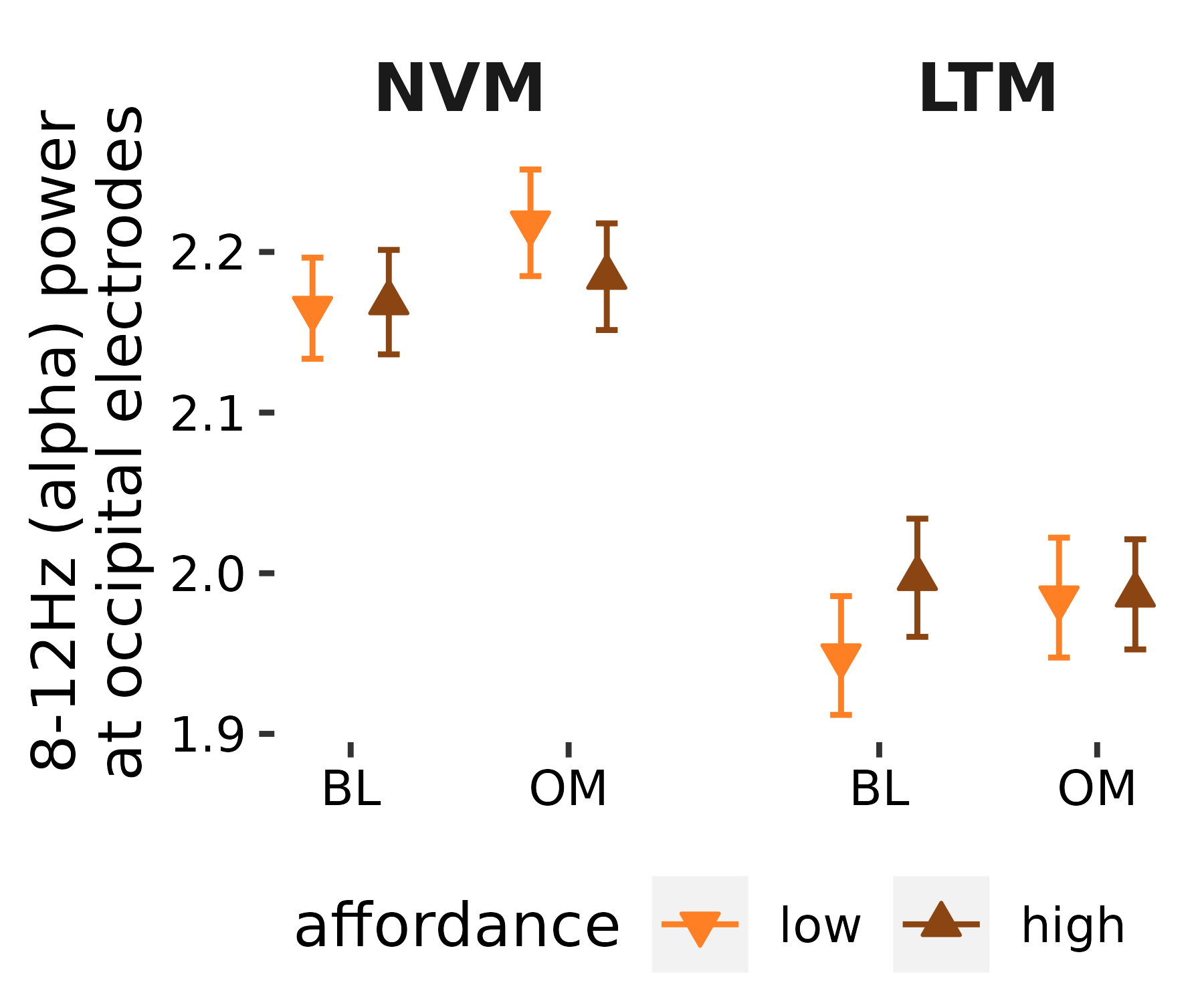


**Supplementary figure 3.** Effect of experimental manipulations on parieto-occipital alpha power. Error bars are within-subject standard errors of the means.


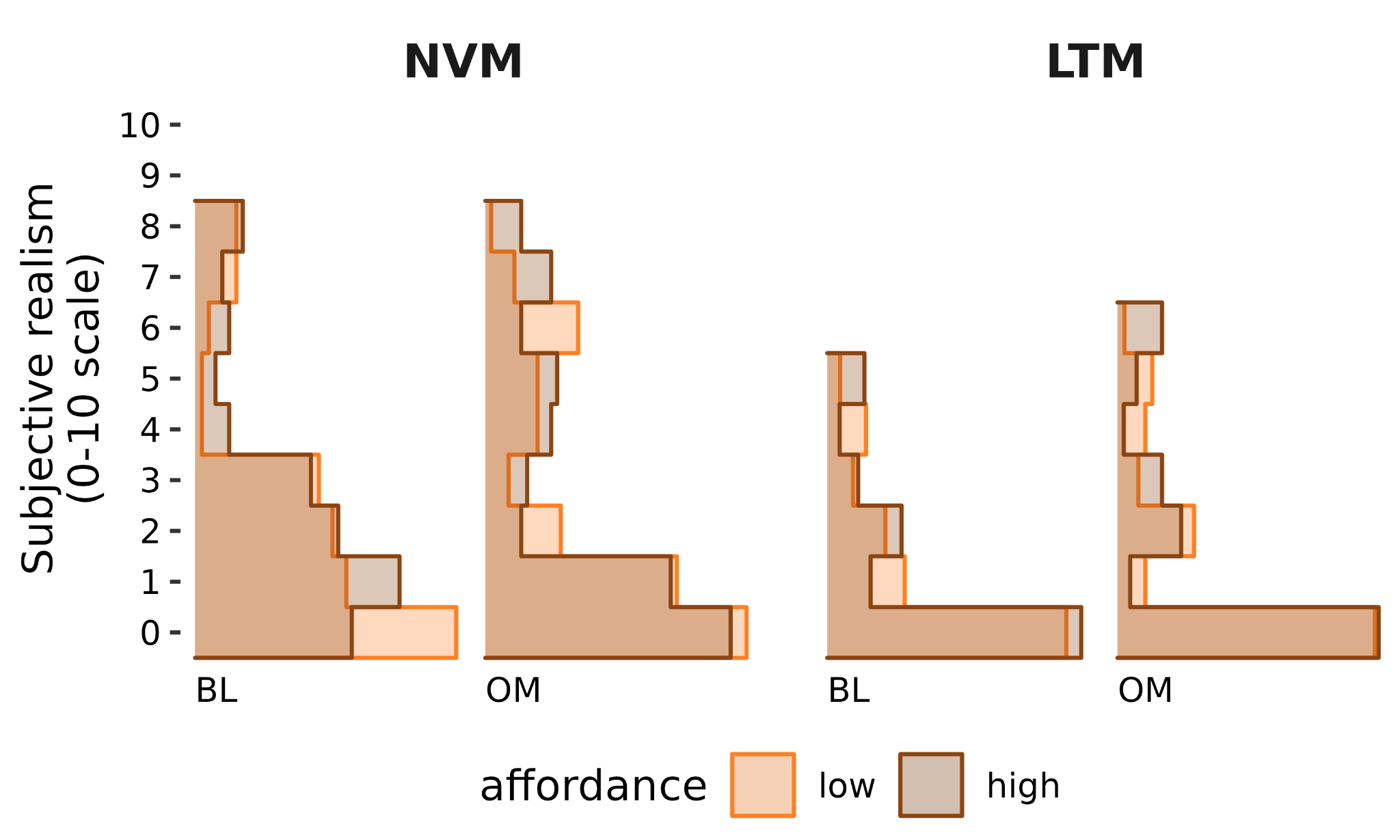


**Supplementary figure 4.** Full distributions of subjective realism ratings. While an effect of affordance has been found across all conditions, it differs qualitatively between groups and states.


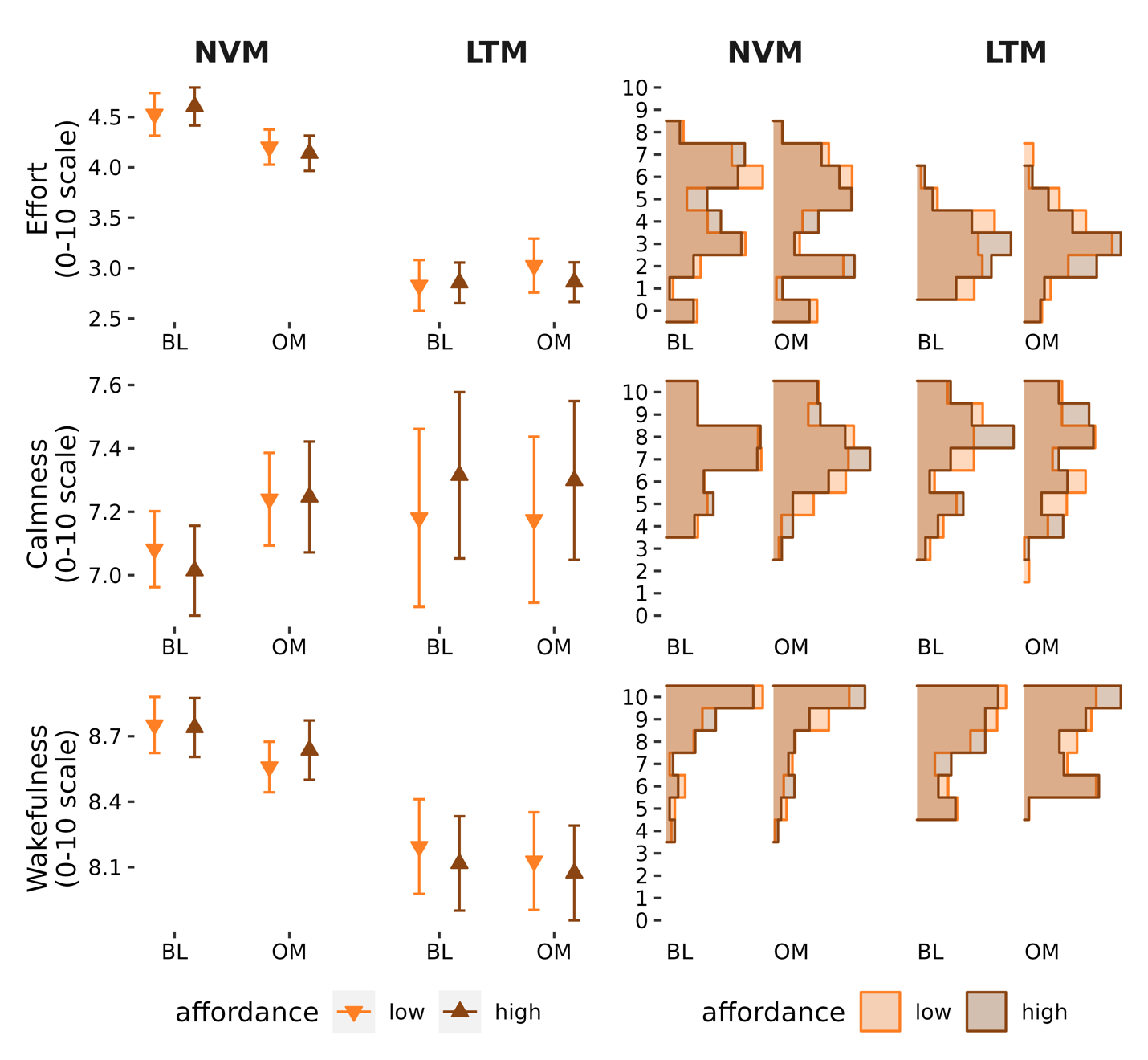


**Supplementary Figure 5.** Differences between groups and conditions (left) and full distributions of ratings (right) for subjective sense of effortfulness, calmness and arousal/wakefulness during the task.
